# The Cognitive Effects of Statins are Modified by Age

**DOI:** 10.1038/s41598-020-63035-2

**Published:** 2020-04-10

**Authors:** Ahmed M. Alsehli, Gaia Olivo, Laura E. Clemensson, Michael J. Williams, Helgi B. Schiöth

**Affiliations:** 1 Uppsala University, Department of Neuroscience, Functional Pharmacology Unit, Uppsala Biomedical Center (BMC), Husargatan 3 (visiting address), Box 593 (postal address), 75124 Uppsala, Sweden; 2 King Abdulaziz University, Department of Physiology, Faculty of Medicine, Al Ehtifalat St., 21589 Jeddah, Kingdom of Saudi Arabia; 3 Sechenov First Moscow State Medical University, Institute for Translational Medicine and Biotechnology, Sechenov Biomedical Science & Technology Park, Trubetskay Str. 8, 119991 Moscow, Russia

**Keywords:** Drug safety, Cognitive neuroscience, Learning and memory, Population screening, Neurology

## Abstract

To reveal new insights into statin cognitive effects, we performed an observational study on a population-based sample of 245,731 control and 55,114 statin-taking individuals from the UK Biobank. Cognitive performance in terms of reaction time, working memory and fluid intelligence was analysed at baseline and two follow-ups (within 5–10 years). Subjects were classified depending on age (up to 65 and over 65 years) and treatment duration (1–4 years, 5–10 years and over 10 years). Data were adjusted for health- and cognition-related covariates. Subjects generally improved in test performance with repeated assessment and middle-aged persons performed better than older persons. The effect of statin use differed considerably between the two age groups, with a beneficial effect on reaction time in older persons and fluid intelligence in both age groups, and a negative effect on working memory in younger subjects. Our analysis suggests a modulatory impact of age on the cognitive side effects of statins, revealing a possible reason for profoundly inconsistent findings on statin-related cognitive effects in the literature. The study highlights the importance of characterising modifiers of statin effects to improve knowledge and shape guidelines for clinicians when prescribing statins and evaluating their side effects in patients.

## Introduction

Statins are the first-choice treatment against hypercholesterolemia and associated cardiovascular disease^1^, the main global cause of morbidity and mortality (World Health Organization). Statins are among the most prescribed drugs worldwide with an estimated 25% of the world population older than 65 years currently under statin treatment and the numbers increasing^2^.

Currently, there is a large controversy about whether or not statins affect cognitive function^3^. Studies have provided indications for both sides, as well as reported beneficial and detrimental effects^4^. Altogether, findings in the literature are highly inconsistent.

In a 2015 review addressing the inconsistency of study outcomes on statin-related cognitive effects, a range of limitations of previous studies has been pointed out, and necessities for future investigations to reveal more conclusive results have been suggested^3^. In this comprehensive review, the authors specifically stated that valid information on the impact of statin exposure during midlife as well as the effect of long-term exposure on cognitive performance is lacking.

On this basis, we hypothesised that statin effects on cognition are not uniform, and that the inconsistency in the literature is related to modulatory factors that might act interactively. The primary aim of our study was to assess, if the effect of statins on cognitive function is dependent on age, by analysing data from a large, population-based cohort. Under this aim, a possible effect of treatment duration was also assessed. The outcomes of our study might thereby add significantly to the current understanding of statin-related cognitive effects.

## Results

**Figure 1:**
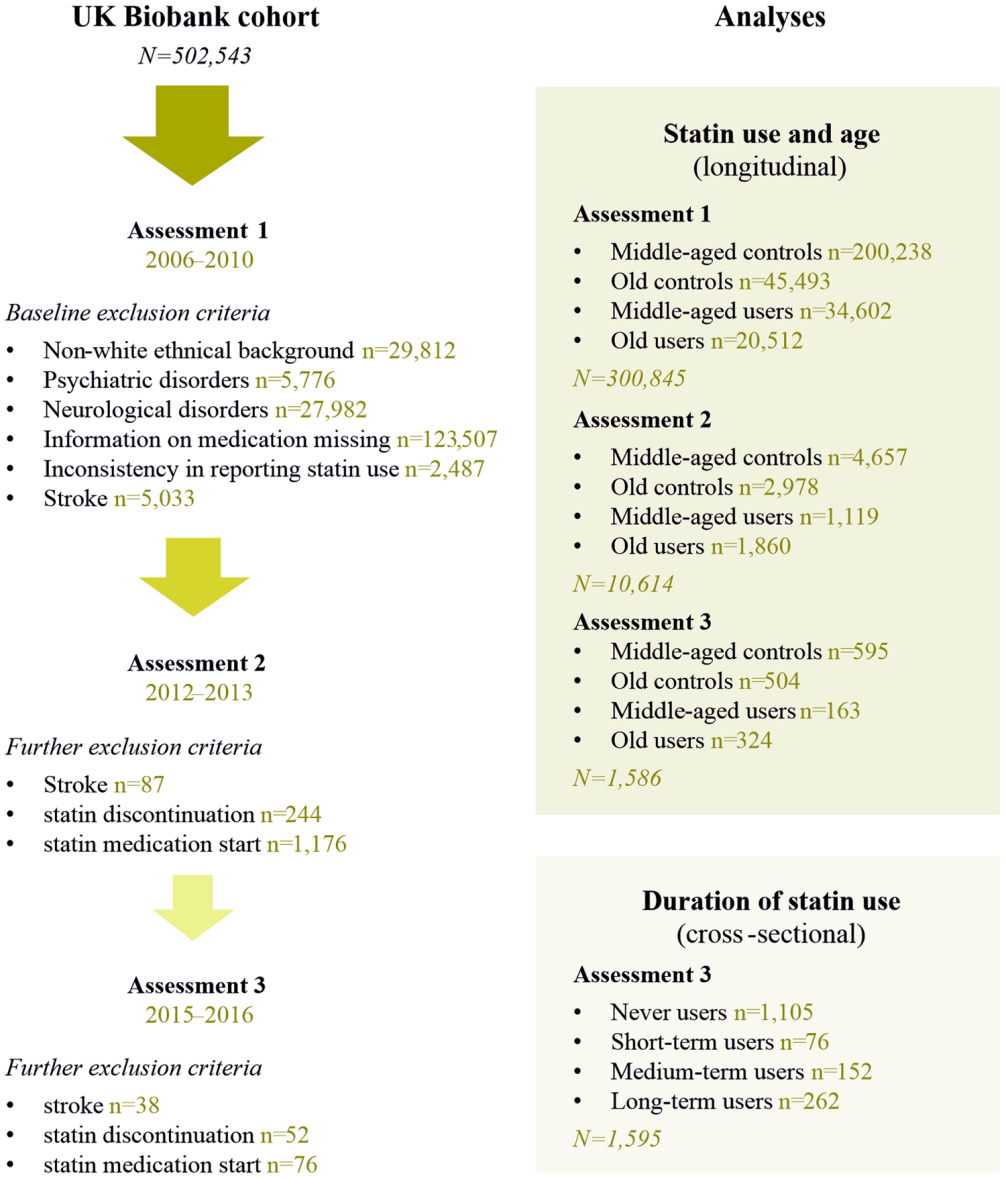
Study design. The figure shows the inclusion/exclusion criteria and categorisation of eligible participants for the different analyses.

An overview over the study design is given in Fig. 1.

### Differences in health-related parameters between controls and statin users

As expected, statin users differed significantly from controls in health-related parameters (Table 1). In fact, mean BMI was significantly higher in statin users than controls (1.4–2.5 kg/m^2^ higher), while the statin group contained a considerably higher incidence of diabetes (12.0–20.2% more people), angina (6.1–15.5% more people), heart attack (5.9–11.8% more people) and hypertension (23.6–36.4% more people). Also, statin-taking participants used insulin (1.5–5.5% more people) and antihypertensive medication (27.0–43.4% more people) more often. Other parameters that differed in statin users, which might affect cognitive readouts, were a moderately lower level of physical activity (0.1–0.2 days less per week), slightly longer mean sleep duration (0.1–0.2 hours per night longer), a moderately higher proportion of current (−0.8–2.0% more) and markedly higher proportion of previous smokers (5.5–10.5% more), higher sub-cohort age (0.4–4.2 years older) and a higher proportion of men in the statin compared to the control group (19.6–26.6% more).

The combined influence of a different health status was further addressed in the following analysis by considering them as covariates.

**Table 1:** Health- and cognition-related characteristics of the study cohort.

	Middle-aged		Old	
	Control	Statin	Control	Statin
**Assessment 1**				
***N***	200,238	34,602	45,493	20,512
***Age, years***	54.2 (6.9)	58.4 (5.2)***	66.9 (1.5)	67.0 (1.5)***
***BMI, kg/m***^***2***^	27.2 (4.8)	29.5 (5.1)***	27.2 (4.3)	28.7 (4.3)***
***Physical activity, days/week***	3.5 (2.3)	3.4 (2.3)***	4.0 (2.3)	3.8 (2.3)***
***Sleep duration, hours***	7.1 (1.1)	7.2 (1.2)***	7.3 (1.1)	7.4 (1.2)***
***Sex, men***	0.371	0.613**	0.414	0.610**
***Education, degree***	0.336	0.271**	0.229	0.197**
***Diabetes***	0.019	0.221**	0.032	0.188**
***Heart attack***	0.005	0.105**	0.014	0.132**
***Angina***	0.008	0.132**	0.024	0.179**
***Hypertension***	0.234	0.598**	0.356	0.624**
***Cholesterol medication***	0.026	1.000**	0.068	1.000**
***Hypertension medication***	0.159	0.593**	0.298	0.343**
***Insulin***	0.004	0.049**	0.006	0.032**
***Alcohol intake, score***	3.115	3.098***	3.072	3.084***
***Smoking status, current***	0.105	0.119**	0.067	0.081**
***Smoking status, ever***	0.336	0.427**	0.422	0.501**
**Assessment 2**				
***N***	4,657	1,119	2,978	1,860
***Age, years***	56.8 (5.6)	59.6 (4.4)***	68.4 (2.7)	68.8 (2.8)***
***BMI, kg/m***^***2***^	26.7 (4.8)	29.2 (5.1)***	26.5 (4.3)	28.4 (4.3)***
***Physical activity, days/week***	3.5 (2.3)	3.4 (2.3)	3.4 (2.3)	3.6 (2.3)**
***Sleep duration, hours***	7.2 (1.0)	7.2 (1.1)	7.3 (1.1)	7.4 (1.1)**
***Sex, men***	0.347	0.611**	0.410	0.678**
***Education, degree***	0.490	0.409**	0.368	0.344
***Diabetes***	0.022	0.229**	0.036	0.178**
***Heart attack***	0.002	0.082**	0.007	0.113**
***Angina***	0.004	0.097**	0.013	0.128**
***Hypertension***	0.227	0.585**	0.333	0.596**
***Cholesterol medication***	0.015	1.000**	0.034	1.000**
***Hypertension medication***	0.163	0.564**	0.285	0.636**
***Insulin***	0.004	0.052**	0.004	0.023**
***Alcohol intake, score***	3.049	3.110***	3.139	3.242***
***Smoking status, current***	0.040	0.032	0.032	0.038
***Smoking status, ever***	0.323	0.386**	0.410	0.479**
**Assessment 3**				
***N***	595	163	504	324
***Age, years***	57.1 (5.2)	60.2 (4.1)**	69.1 (3.1)	70.2 (3.4)**
***BMI, kg/m***^***2***^	26.6 (4.8)	29.0 (5.7)**	26.1 (4.2)	27.5 (4.0)**
***Physical activity, days/week***	3.5 (2.2)	3.5 (2.2)	4.1 (2.1)	4.0 (2.2)
***Sleep duration, hours***	7.1 (1.0)	7.2 (1.1)	7.2 (1.1)	7.4 (1.0)**
***Sex, men***	0.324	0.576**	0.414	0.680**
***Education, degree***	0.548	0.472	0.437	0.448
***Diabetes***	0.026	0.213**	0.039	0.159**
***Heart attack***	0.002	0.061**	0.006	0.104**
***Angina***	0	0.061**	0.004	0.104**
***Hypertension***	0.255	0.491**	0.313	0.557**
***Cholesterol medication***	0.010	1.000**	0.022	1.000**
***Hypertension medication***	0.209	0.479**	0.301	0.590**
***Insulin***	0.005	0.060**	0.003	0.018
***Alcohol intake, score***	2.905	3.121	3.217	3.172
***Smoking status, current***	0.035	0.055	0.010	0.028
***Smoking status, ever***	0.325	0.370	0.427	0.449

The table lists the health- and cognition-related parameters of controls (no statin use at the respective assessment) and statin users (statin use at the respective assessment) for each assessment (1–3) and the two age groups analysed (middle-aged: up to 65 years, and old: over 65 years). The values refer to mean and standard deviation (in parentheses) for physiological parameters, and proportions for frequencies and incidences. N = number of subjects. Physiological parameters between statin users and controls within an age group have been analysed using multiple t-tests and multiple comparison correction (*P < 0.013, **P < 0.01, ***P < 0.001), frequencies and incidences were compared using the Fisher’s exact test and multiple comparison adjustment (*P < 0.005, **P < 0.001) and the alcohol intake scores were compared using the Chi-square test (*P < 0.05, **P < 0.01, ***P < 0.001).

### Effects of health-related parameters on cognitive performance in controls and statin users

In order to rule out confounding through differences in the health status of controls and statin users, we investigated the effect of the above-mentioned 15 health-related parameters (Table 1) on the performance readouts of three cognitive tests. For all following analyses, the parameters that showed significant effects on either of the cognitive readouts, and in either controls or statin users, were therefore included as covariates (the statistically significant test results are shown in Table 2).

**Table 2:** Covariates of cognitive performance.

Reaction time				
Controls	**B**	**SE**	**CI 95%**	***P***
***Age***	0.008	0.0003	0.007, 0.009	<0.001***
***Physical activity***	0.003	0.0010	0.001, 0.005	=0.001**
***Sleep duration***	0.007	0.0023	0.003, 0.012	=0.001**
***Sex (male vs female)***	0.028	0.0005	0.033, 0.019	<0.001***
***Education (degree vs no degree)***	−0.035	0.0045	−0.044, −0.026	<0.001***
***Hypertension (yes vs no)***	0.017	0.0051	0.008, 0.027	=0.001**
***Cholesterol medication (non-statin) (yes vs no)***	0.053	0.0156	0.023, 0.084	=0.001**
***Hypertension medication (yes vs no)***	0.022	0.0054	0.012, 0.033	<0.001***
***Alcohol intake (daily/almost daily vs never)***	−0.035	0.0101	−0.055, −0.015	=0.001**
***Alcohol intake (3–4 times per week vs never)***	−0.044	0.0097	−0.063, −0.025	<0.001***
***Alcohol intake (1–2 times per week vs never)***	−0.038	0.0097	−0.057, −0.019	<0.001***
***Alcohol intake (1–3 times per month vs never)***	−0.037	0.0108	−0.058, −0.016	=0.001**
***Alcohol intake (special occasions vs never)***	−0.026	0.0112	−0.048, −0.004	=0.020*
Statin users	**B**	**SE**	**CI 95%**	***P***
***Age***	0.007	0.0007	0.005, 0.008	<0.001***
***Sex (male vs female)***	0.030	0.0056	0.011, 0.048	=0.002**
***Education (degree vs no degree)***	0.042	0.0093	0.024, 0.060	<0.001***
***Alcohol intake (daily/almost daily)***	−0.040	0.0189	−0.077, −0.003	=0.032*
***Alcohol intake (3–4 times per week)***	−0.049	0.0179	−0.085, −0.014	=0.006**
***Alcohol intake (1–3 times per month)***	−0.050	0.0204	−0.090, −0.010,	=0.014*
**Working memory**				
Controls	**B**	**SE**	**CI 95%**	***P***
***Age***	0.017	0.0012	0.014, 0.019	<0.001***
***BMI***	−0.005	0.0020	−0.009, −0.001	=0.015*
***Physical activity***	0.013	0.0042	0.005, 0.022	=0.001**
***Sex (male vs female)***	−0.071	0.0190	−0109, −0.034	<0.001***
***Education (degree vs no degree)***	−0.077	0.0184	−0.113. −0.041	<0.001***
***Hypertension (yes vs no)***	0.041	0.0202	0.001, 0.081	=0.043*
Statin users	**B**	**SE**	**CI 95%**	***P***
***Age***	0.011	0.0039	0.003, 0.019	=0.006**
***BMI***	−0.008	0.0038	−0.016, −0.001	=0.028*
***Education (degree vs no degree)***	−0.121	0.0394	−0.198, −0.044	=0.002**
***Hypertension (yes vs no)***	1.411	0.023	1.365, 1.457	<0.001***
**Fluid intelligence**				
Controls	**B**	**SE**	**CI 95%**	***P***
***Age***	−0.038	0.0034	−0.045, −0.035	<0.001***
***Physical activity***	−0.098	0.0110	−0.076, −0.119	<0.001***
***Sleep duration***	−0.054	0.0237	−0.007, −0.100,	=0.024*
***Sex (male vs female)***	−0.188	0.0525	−0.291, −0.085	<0.001***
***Education (degree vs no degree)***	1.248	0.0489	1.344, 1.152	<0.001***
***Angina (yes vs no)***	−0.580	0.2672	−1.103, −0.056	=0.030*
***Cholesterol medication (non-statin) (yes vs no)***	−0.503	0.1543	0.805, 0.201	=0.001**
***Alcohol intake (daily/almost daily)***	0.458	0.1121	0.329, 0.678	<0.001***
***Alcohol intake (3–4 times per week)***	0.461	0.1068	0.251, 0.670	<0.001***
***Alcohol intake (1–3 times per month)***	0.351	0.1195	0.117, 0.585	=0.003**
Statin users	**B**	**SE**	**CI 95%**	***P***
***Age***	−0.066	0.0098	−0.085, −0.047	<0.001***
***Physical activity***	−0.087	0.0238	−0.041, −0.134	<0.001***
***Education (degree vs no degree)***	−1.284	0.0909	−1.496, −1.072	<0.001***
***Heart attack (yes vs no)***	−0.315	0.1593	−0.627, −0.002	=0.048*
***Alcohol intake (daily/almost daily)***	0.509	0.2303	0.058, 0.961	=0.027*

Statistical test results for significant effects of health-related parameters (Table 1) on the cognitive performance of controls and statin users in the three cognitive tests measuring reaction time (card game “Snap”), working memory (“Pairs matching test”) and reasoning abilities (“Fluid intelligence test”). Generalised linear models have been used for statistical analysis. B = regression coefficient, SE = standard error, CI 95% = 95% confidence interval (min and max). *P < 0.05, **P < 0.01, ***P < 0.001.

For reaction time, we found modulatory effects of age, sex, education and alcohol intake frequency in both controls and statin users as well as additional effects of physical activity, sleep duration, hypertension, antihypertensive medication and non-statin cholesterol-lowering medication in controls.

For working memory, we found effects of age, education, BMI and hypertension in both controls and statin users as well as additional effects of sex and physical activity in controls.

For fluid intelligence, we found an influence of age, education, physical activity and alcohol intake frequency in both controls and statin users as well as additional effects of sex, sleep duration, angina and non-statin cholesterol lowering medication in controls and heart attack in statin users.

### General performance in the three tests

**Figure 2:**
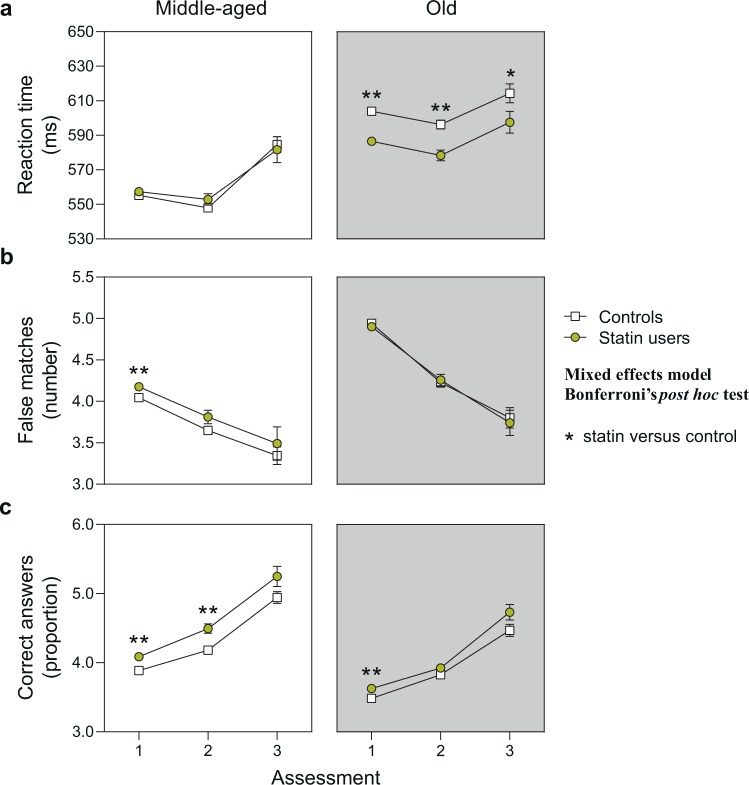
Cognitive performance of middle-aged and old control persons and statin users. The graph shows the longitudinal cognitive performance over the three assessments. Middle-aged: persons up to 65 years of age; old: persons over 65 years of age. (**a**) Average reaction time in the card game “Snap”. Estimated marginal means and standard error with continuous predictors fixed at alcohol = 3, sleep duration = 7, physical activity = 4, qualification = 0 and smoking = 1. Total n(included) = 291,947. (**b**) Average number of mismatches in the “Pairs matching test” relating to working memory function. Estimated marginal means and standard error with continuous predictors fixed at BMI = 27.51, physical activity = 4 and qualification = 0. Total n(included) = 289,091. (**c**) Number of correct answers per attempted questions in the “Fluid intelligence test” measuring reasoning abilities. Estimated marginal means and standard error with continuous predictors fixed at alcohol = 3, sleep duration = 7, physical activity = 4 and qualification = 0. Total n(included) = 101,436. *P < 0.017, **P < 0.01, ***P < 0.001.

The general performance in the three tests in terms of training effect (repeated assessments) or effect of advanced age (middle-aged versus old) was similar in controls and statin users (Fig. 2, individual statistical results for controls and statin users are given in Table 3).

#### Training effect

Participants showed a continuous improvement with repeated testing in the working memory (training effect: F_(2, 241,368)_ = 38.82, *P* < 0.001 in middle-aged and F_(2, 71,665)_ = 126.5, *P* < 0.001 in old subjects) and reasoning test (training effect: F_(2, 241,368)_ = 3.589, *P* = 0.028 in middle-aged and F_(2, 71,665)_ = 17.34, *P* < 0.001 in old subjects), and a less consistent training effect on reaction time (training effect: F_(2, 241,368)_ = 1.872, *P* = 0.1538 in middle-aged and F_(2, 71,665)_ = 3.718, *P* = 0.024 in old subjects) (Fig. 2a–c).

#### Age effect

On the other hand, advanced age had a generally negative impact on test performance: older subjects had a longer reaction time (age effect: F_(1, 300,841)_ = 629.2, *P* < 0.001 at the first assessment, F_(1, 10,610)_ = 245.2, *P* < 0.001 at the second assessment and F_(1, 1,582)_ = 17.92, *P* < 0.001 at the third assessment) (Fig. 2a), made more mistakes in the working memory test (age effect: F_(1, 300,841)_ = 2,480, *P* < 0.001 at the first assessment, F_(1, 10,610)_ = 66.93, *P* < 0.001 at the second assessment and F_(1, 1,582)_ = 2,155, *P* < 0.001 at the third assessment) (Fig. 2b), and provided fewer correct answers when assessing fluid intelligence (age effect: F_(1, 300,841)_ = 56.38, *P* < 0.001 at the first assessment, F_(1, 10,610)_ = 65.05, *P* < 0.001 at the second assessment and F_(1, 1,582)_ = 7,686, *P* < 0.001 at the third assessment) (Fig. 2c).

**Table 3:** General effect of training and age on cognitive performance.

Reaction time				
**Training effect**	**MD**	**SE**	**CI 95%**	***P***
***Controls: middle-aged, assessment 1 vs assessment 2***	7.356	5.381	−5.493, 20.20	=0.432
***Controls: middle-aged, assessment 1 vs assessment 3***	−29.33	14.90	−64.92, 6.260	=0.140
***Controls: middle-aged, assessment 2 vs assessment 3***	−36.68	15.80	−74.42, 1.053	=0.060
***Controls: old, assessment 1 vs assessment 2***	7.065	3.858	−2.148, 16.28	=0.188
***Controls: old, assessment 1 vs assessment 3***	−9.577	9.136	−31.39, 12.24	=0.649
***Controls: old, assessment 2 vs assessment 3***	−16.64	9.825	−40.10, 6.817	=0.247
***Statin users: middle-aged, assessment 1 vs assessment 2***	4.416	11.03	−21.91, 30.74	=0.970
***Statin users: middle-aged, assessment 1 vs assessment 3***	−24.36	28.50	−92.41, 43.70	=0.776
***Statin users: middle-aged, assessment 2 vs assessment 3***	−28.78	30.44	−101.4, 43.90	=0.718
***Statin users: old, assessment 1 vs assessment 2***	7.545	4.939	−4.249, 19.34	=0.334
***Statin users: old, assessment 1 vs assessment 3***	−10.02	11.42	37.29, 17.26	=0.762
***Statin users: old, assessment 2 vs assessment 3***	−17.56	12.28	−46.88, 11.76	=0.392
**Age effect**	**MD**	**SE**	**CI 95%**	***P***
**Controls: assessment 1, middle-aged vs old**	−52.48	1.762	−56.42, −48.54	<0.001**
**Controls: assessment 2, middle-aged vs old**	−52.77	2.834	−59.11, −46.43	<0.001**
**Controls: assessment 3, middle-aged vs old**	−32.72	6.669	−47.65, −17.80	<0.001**
**Statin users: assessment 1, middle-aged vs old**	−34.56	2.989	−41.25, −27.88	<0.001**
**Statin users: assessment 2, middle-aged vs old**	−31.43	4.570	−41.66, −21.21	<0.001**
**Statin users: assessment 3, middle-aged vs old**	−20.22	10.58	−43.90, 3.462	=0.109
**Working memory**				
**Training effect**	**MD**	**SE**	**CI 95%**	***P***
***Controls: middle-aged, assessment 1 vs assessment 2***	0.397	0.046	0.287, 0.507	<0.001**
***Controls: middle-aged, assessment 1 vs assessment 3***	0.700	0.127	0.396, 1.004	<0.001**
***Controls: middle-aged, assessment 2 vs assessment 3***	0.303	0.135	−0.019, 0.625	=0.072
***Controls: old, assessment 1 vs assessment 2***	0.715	0.064	0.562, 0.868	<0.001**
***Controls: old, assessment 1 vs assessment 3***	1.140	0.152	0.778, 1.502	<0.001**
***Controls: old, assessment 2 vs assessment 3***	0.425	0.163	0.036, 0.814	=0.027
***Statin users: middle-aged, assessment 1 vs assessment 2***	0.365	0.094	0.140, 0.590	=0.001*
***Statin users: middle-aged, assessment 1 vs assessment 3***	0.687	0.243	0.106, 1.268	=0.014
***Statin users: middle-aged, assessment 2 vs assessment 3***	0.322	0.260	−0.298, 0.942	=0.517
***Statin users: old, assessment 1 vs assessment 2***	0.644	0.082	0.448, 0.840	<0.001**
***Statin users: old, assessment 1 vs assessment 3***	1.161	0.190	0.709, 1.613	<0.001**
***Statin users: old, assessment 2 vs assessment 3***	0.517	0.204	0.031, 1.003	=0.033
**Age effect**	**MD**	**SE**	**CI 95%**	***P***
**Controls: assessment 1, middle-aged vs old**	−0.896	0.017	−0.933, −0.859	<0.001**
**Controls: assessment 2, middle-aged vs old**	−0.578	0.066	−0.725, −0.431	<0.001**
**Controls: assessment 3, middle-aged vs old**	−0.456	0.008	−0.474, −0.438	<0.001**
**Statin users: assessment 1, middle-aged vs old**	−0.724	0.028	−0.787, −0.661	<0.001**
**Statin users: assessment 2, middle-aged vs old**	−0.445	0.106	−0.683, −0.207	<0.001**
**Statin users: assessment 3, middle-aged vs old**	−0.250	0.013	−0.279, −0.221	<0.001**
**Fluid intelligence**				
**Training effect**	**MD**	**SE**	**CI 95%**	***P***
***Controls: middle-aged, assessment 1 vs assessment 2***	−0.294	0.179	−0.722, 0.134	=0.273
***Controls: middle-aged, assessment 1 vs assessment 3***	−1.057	0.496	−2.241, 0.127	=0.096
***Controls: middle-aged, assessment 2 vs assessment 3***	−0.763	0.526	−2.019, 0.493	=0.379
***Controls: old, assessment 1 vs assessment 2***	−0.340	0.113	−0.610, −0.070	=0.008
***Controls: old, assessment 1 vs assessment 3***	−0.985	0.268	−1.625, −0.345	=0.001*
***Controls: old, assessment 2 vs assessment 3***	−0.645	0.288	−1.333, 0.043	=0.074
***Statin users: middle-aged, assessment 1 vs assessment 2***	−0.408	0.367	−1.284, 0.468	=0.605
***Statin users: middle-aged, assessment 1 vs assessment 3***	−1.163	0.949	−3.428, 1.102	=0.526
***Statin users: middle-aged, assessment 2 vs assessment 3***	−0.755	1.013	−3.174, 1.664	=0.839
***Statin users: old, assessment 1 vs assessment 2***	−0.298	0.145	−0.644, 0.048	=0.115
***Statin users: old, assessment 1 vs assessment 3***	−1.103	0.335	−1.903, −0.303	=0.003
***Statin users: old, assessment 2 vs assessment 3***	−0.805	0.360	−1.665, 0.055	=0.074
**Age effect**	**MD**	**SE**	**CI 95%**	***P***
**Controls: assessment 1, middle-aged vs old**	0.402	0.058	0.272, 0.532	<0.001**
**Controls: assessment 2, middle-aged vs old**	0.356	0.060	0.221, 0.491	<0.001**
**Controls: assessment 3, middle-aged vs old**	0.474	0.006	0.461, 0.488	<0.001**
**Statin users: assessment 1, middle-aged vs old**	0.458	0.099	0.237, 0.679	<0.001**
**Statin users: assessment 2, middle-aged vs old**	0.568	0.097	0.350, 0.786	<0.001**
**Statin users: assessment 3, middle-aged vs old**	0.518	0.010	0.497, 0.539	<0.001**

Statistical test results from the analysis of training (repeated assessment) and age (middle-aged versus old) effects of controls and statin users in the three cognitive tests measuring reaction time (card game “Snap”), working memory (“Pairs matching test”) and reasoning abilities (“Fluid intelligence test”), adjusted for covariates (r*eaction time*: sex, age, education, physical activity, sleep duration, alcohol intake, smoking status, angina, hypertension, cholesterol-lowering and anti-hypertension medication; *working memory*: sex, age, education, BMI, physical activity and hypertension; *fluid intelligence*: sex, age, education, physical activity, sleep duration, alcohol intake, heart attack, angina and cholesterol-lowering medication). Two-way ANOVAs and multiple comparison correction have been used for statistical analysis. MD = mean difference, SE = standard error, CI 95% = 95% confidence interval (min and max). *P < 0.003, **P < 0.001 for training effects and *P < 0.006, **P < 0.001 for age effects.

### Effects of statin use and age on cognitive performance over time

The use of statin medication significantly affected the performance in all three cognitive tests, however, in a differential manner (individual test results with statistical outcomes are described below). Statin effects were generally weaker or absent at follow-up, most likely due to the lower sample number and resulting higher variation rather than due to a true effect change. User age significantly influenced the effect of statins on cognitive performance.

#### Reaction time

Statin use significantly affected reaction time (main statin effect: F_(1, 291,934)_ = 10.655, *P* = 0.001) with statin users performing overall better than controls (Fig. 2a; pairwise contrasts are given in Table 4). The positive effect was detectable at all three assessments, although a significant statin*time interaction effect (F_(4, 291,930)_ = 28.725, *P* < 0.001) and following *post hoc* analysis suggested that the effect lost strength with repeated assessment, possibly due to lower sample size and thus higher variation at follow-up (assessment 1: F_(1, 291,930)_ = 8.491, *P* = 0.004; assessment 2: F_(1, 291,930)_ = 7.132, *P* = 0.008; assessment 3: F_(1, 291,930)_ = 6.601, *P* = 0.010). We also detected a significant statin*time*age interaction effect (F_(9, 291,925)_ = 38.765, *P* < 0.001), and *post hoc* testing indicated that statin effects are not similar among the age groups. Specifically, the positive effect of statin use on reaction time was only present in old statin users (assessment 1: F_(1, 291,925)_ = 85.870, *P* < 0.001; assessment 2: F_(1, 291,925)_ = 7.132, *P* < 0.001; assessment 3: F_(1, 291,925)_ = 6.601, *P* = 0.046), while no difference in performance was observed for middle-aged statin users (assessment 1: F_(1, 291,925)_ = 1.880, *P* = 0.170; assessment 2: F_(1, 291,925)_ = 1.677, *P* = 0.195; assessment 3: F_(1, 291,925)_ = 3.982, *P* = 0.739).

#### Working memory

Statin treatment had a different effect on longitudinal working memory function (Fig. 2b; pairwise contrasts are given in Table 4). Statin use overall significantly impaired test performance (main statin effect: F_(1, 289,083) = _14.330, *P* < 0.001). Again, there was a significant statin*time interaction (F_(4, 289,079)_ = 102.523, *P* < 0.001) with weakening of the effect at the second and third assessment, this time only reaching statistical significance at the first assessment (assessment 1: F_(1, 289,079)_ = 20.840, *P* < 0.001; assessment 2: F_(1, 289,079)_ = 0.068, *P* = 0.794; assessment 3: F_(1, 289,079)_ = 0.289, *P* = 0.591). Statin effects differed between the two age groups (statin*time*age effect F_(9, 289,074)_ = 49.716, *P* < 0.001) with the negative effect on working memory performance only being present in middle-aged (assessment 1: F_(1, 289,074)_ = 52.441, *P* < 0.001; assessment 2: F_(1, 289,074)_ = 3.238, *P* = 0.072; assessment 3: F_(1, 289,074)_ = 0.398, *P* = 0.528) but not old persons (assessment 1: F_(1, 289,074)_ = 1.862, *P* = 172; assessment 2: F_(1, 289,074)_ = 0.127, *P* = 0.722; assessment 3: F_(1, 289,074)_ = 0.096, *P* = 0.756).

#### Fluid intelligence

Statin users exhibited overall improved reasoning abilities compared to controls (F_1, 101,424_ = 29.841, *P* < 0.001) (Fig. 2c; pairwise contrasts are given in Table 4). The positive influence of statin treatment was detectable at all assessments (assessment 1: F_(1, 101,420)_ = 18.668, *P* < 0.001; assessment 2: F_(1, 101,420)_ = 10.944, *P* = 0.001; assessment 3: F_(1, 101,420)_ = 4.833, *P* = 0.028), although being weaker at follow-up (statin*time interaction effect F_(4, 101,420)_ = 134.875, *P* < 0.001). In this test, both middle-aged and old statin users benefitted from statin treatment (middle-aged assessment 1: F_(1, 101,416)_ = 21.571, *P* < 0.001; middle-aged assessment 2: F_(1, 101,416)_ = 16.267, *P* < 0.001; middle-aged assessment 3: F_(1, 101,416)_ = 3.318, *P* = 0.069; old assessment 1: F_(1, 101,416)_ = 10.325, *P* = 0.001; old assessment 2: F_(1, 101,416)_ = 2.165, *P* < 0.141; old assessment 3: F_(1, 101,416)_ = 3.335, *P* = 0.068), although the effect was stronger in the younger group, still leading to a significant statin*time*age effect (F_(9, 101,416)_ = 63.428, *P* < 0.001).

**Table 4:** Effect of statin use and age on cognition.

Reaction time *(n* = *291.947)*	CE	SE	CI 95%	*P*
***Statin users vs controls***	−4.330	1.327	−6.931, −1.730	=0.001**
***Assessment 1: all ages, statin users vs controls***	−4.237	1.454	−7.087, −1.387	=0.004**
***Assessment 2: all ages, statin users vs controls***	−7.337	2.747	−12.722, −1.952	=0.008**
***Assessment 3: all ages, statin users vs controls***	−15.001	5.839	−26.446, −3.557	=0.010*
***Assessment 1: middle-aged, statin users vs controls***	2.010	1.466	−0.863, 4.883	=0.170
***Assessment 2: middle-aged, statin users vs controls***	4.950	3.822	−2.542, 12.441	=0.195
***Assessment 3: middle-aged, statin users vs controls***	−2.960	8.886	−20.376, 14.456	=0.739
***Assessment 1: old, statin users vs controls***	−15.904	1.716	−19.268, −12.540	<0.001***
***Assessment 2: old, statin users vs controls***	−16.384	3.725	−23.685, −9.083	<0.001***
***Assessment 3: old, statin users vs controls***	−15.466	7.751	−30.658, −0.275	=0.046
**Working memory** ***(n*** **=** ***289.091)***	**CE**	**SE**	**CI 95%**	***P***
***Statin users vs controls***	0.065	0.017	0.031, 0.099	<0.001***
***Assessment 1: all ages, statin users vs controls***	0.080	0.018	0.046, 0.115	<0.001***
***Assessment 2: all ages, statin users vs controls***	0.018	0.068	−0.116, 0.152	=0.794
***Assessment 3: all ages, statin users vs controls***	−0.085	0.157	−0.393, 0.224	=0.591
***Assessment 1: middle-aged, statin users vs controls***	0.132	0.018	0.096, 0.168	<0.001***
***Assessment 2: middle-aged, statin users vs controls***	0.164	0.091	−0.015, 0.342	=0.072
***Assessment 3: middle-aged, statin users vs controls***	0.145	0.229	−0.305, 0.594	=0.528
***Assessment 1: old, statin users vs controls***	−0.040	0.029	−0.970, 0.017	=0.172
***Assessment 2: old, statin users vs controls***	0.031	0.088	−0.141, 0.203	=0.722
***Assessment 3: old, statin users vs controls***	−0.061	0.196	−0.445, 0.323	=0.756
**Fluid intelligence** ***(n*** **=** ***101.436)***	**CE**	**SE**	**CI 95%**	***P***
***Statin users vs controls***	0.217	0.040	0.139, 0.294	<0.001***
***Assessment 1: all ages, statin users vs controls***	0.173	0.040	0.094, 0.251	<0.001***
***Assessment 2: all ages, statin users vs controls***	0.188	0.057	0.077, 0.299	<0.001***
***Assessment 3: all ages, statin users vs controls***	0.241	0.110	0.026, 0.456	=0.028
***Assessment 1: middle-aged, statin users vs controls***	0.198	0.043	0.114, 0.282	<0.001***
***Assessment 2: middle-aged, statin users vs controls***	0.312	0.077	0.160, 0.463	<0.001***
***Assessment 3: middle-aged, statin users vs controls***	0.304	0.167	−0.023, 0.630	=0.069
***Assessment 1: old, statin users vs controls***	0.142	0.944	0.056, 0.229	<0.001***
***Assessment 2: old, statin users vs controls***	0.101	0.068	−0.033, 0.235	=0.141
***Assessment 3: old, statin users vs controls***	0.259	0.142	−0.019, 0.538	=0.068

Statistical test results from the analysis of performance of controls and statin users in the three cognitive tests measuring reaction time (card game “Snap”), working memory (“Pairs matching test”) and reasoning abilities (“Fluid intelligence test”), adjusted for covariates (r*eaction time*: sex, age, education, physical activity, sleep duration, alcohol intake, smoking status, angina, hypertension, cholesterol-lowering and anti-hypertension medication; *working memory*: sex, age, education, BMI, physical activity and hypertension; *fluid intelligence*: sex, age, education, physical activity, sleep duration, alcohol intake, heart attack, angina and cholesterol-lowering medication). Mixed effects models and correction for multiple comparisons have been applied. CE = contrast estimate, SE = standard error, CI 95% = 95% confidence interval (min and max). *P < 0.017, **P < 0.01, ***P < 0.001.

### Modulatory influence of treatment duration on statin cognitive effects

**Figure 3:**
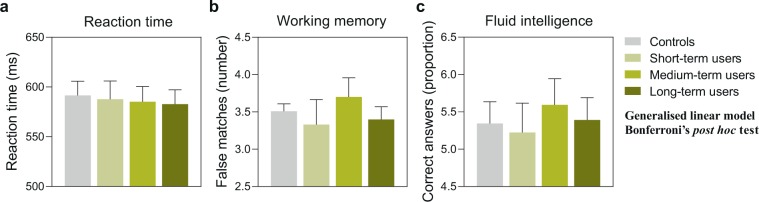
Cognitive performance of statin users with differently long treatment duration compared to controls. The graph shows the cognitive performance at the third assessment. Controls: no statin use; short-term users: 1–4 years of statin use; medium-term users: 5–10 years of statin use; long-term users: >10 years of statin use. (**a**) Average reaction time in the card game “Snap”. Estimated marginal means and standard error with continuous predictors fixed at sleep duration = 7.19 and physical activity = 3.82. Total n(included) = 1,510. (**b**) Average number of mismatches in the “Pairs matching test” relating to short-term memory function. Estimated marginal means and standard error with continuous predictors fixed at BMI = 26.83 and physical activity = 3.81. Total n(included) = 1,480. (**c**) Number of correct answers per attempted questions in the “Fluid intelligence test” measuring reasoning abilities. Estimated marginal means and standard error with continuous predictors fixed at sleep duration = 7.20 and physical activity = 3.82. Total n(included) = 1,477. For all comparisons: *P* > 0.017.

Since we detected a significant modulatory effect of age on the cognitive performance of statin users, the question arose whether this could be based on differently long treatment durations. In order to evaluate this, we performed an additional cross-sectional analysis on the influence of statin treatment duration. We found no difference between statin users classified as short-term, medium-term or long-term users based on statin treatment durations ranging between 1–4 and over 10 years (Fig. 3, Table 5).

**Table 5:** Effect of statin treatment duration on cognition.

Reaction time *(n* = *1,510)*	B	SE	CI 95%	*P*
***Short-term users vs controls***	−0.007	0.044	−0.092, 0.079	=0.880
***Medium-term users vs controls***	−0.011	0.040	−0.089, 0.067	=0.784
***Long-term users vs controls***	−0.015	0.039	−0.092, 0.061	=0.700
***Short-term users vs medium-term users***	0.004	0.025	−0.045, 0.054	=0.866
***Short-term users vs long-term users***	0.008	0.024	−0.039, 0.056	=0.726
***Medium-term users vs long-term users***	0.004	0.017	−0.029, 0.037	=0.803
**Working memory** ***(n* = *1,480)***	**B**	**SE**	**CI 95%**	***P***
***Short-term users vs controls***	−0.051	0.105	−0.256, 0.154	=0.627
***Medium-term users vs controls***	0.055	0.075	−0.093, 0.202	=0.466
***Long-term users vs controls***	−0.030	0.059	−0.145, 0.084	=0.604
***Short-term users vs medium-term users***	−0.106	0.122	−0.345, 0.134	=0.387
***Short-term users vs long-term users***	−0.021	0.112	−0.240, 0.199	=0.854
***Medium-term users vs long-term users***	0.085	0.083	−0.078, 0.248	=0.306
**Fluid intelligence** ***(n* = *1,477)***	**B**	**SE**	**CI 95%**	***P***
***Short-term users vs controls***	−0.488	0.596	−1.655, 0.679	=0.413
***Medium-term users vs controls***	0.507	0.516	−0.504, 1.518	=0.325
***Long-term users vs controls***	0.110	0.506	−0.882, 1.103	=0.828
***Short-term users vs medium-term users***	−0.371	0.339	−1.034, 0.293	=0.274
***Short-term users vs long-term users***	−0.167	0.313	−0.780, 0.447	=0.594
***Medium-term users vs long-term users***	0.204	0.252	−0.290, 0.698	=0.419

Statistical test results from the analysis of performance of controls, short-term statin users (1–4 years), medium-term statin users (5–10 years) and long-term statin users (>10 years) at the third assessment in the three cognitive tests measuring reaction time (card game “Snap”), working memory (“Pairs matching test”) and reasoning abilities (“Fluid intelligence test”), adjusted for covariates (r*eaction time*: sex, age, education, physical activity, sleep duration, alcohol intake, smoking status, angina, hypertension, cholesterol-lowering and anti-hypertension medication; *working memory*: sex, age, education, BMI, physical activity and hypertension; *fluid intelligence*: sex, age, education, physical activity, sleep duration, alcohol intake, heart attack, angina and cholesterol-lowering medication). Generalised linear models and correction for multiple comparisons have been applied. B = regression coefficient, SE = standard error, CI 95% = 95% confidence interval (min, max). The threshold for significance was set at *P* < 0.017.

## Discussion

Previous studies have been inconsistent as to whether a potential association between statins use and cognitive abilities exists. Also, consensus has not been reached regarding the potential usefulness of statins in the prevention of dementia or Alzheimer disease^4^. The large discrepancies in the outcomes of different studies have been suggested to be due to limitations in the sample analysed. In particular, data on younger statin users or longer treatment durations are lacking, which would enable the assessment of a specific effect of age on statin cognitive effects^3^. In regard to this, we performed an observational study, incorporating a large set of population-based data, which allowed for the specific analysis of a possible modulatory influence of subject age on statin-related cognitive effects. Such a modifying effect might contribute significantly to the variable outcomes of published studies, which include wide variations in subject age^5–11^. In fact, we identified that statin effects varied in quality, strength and appearance depending on user age. Specifically, statins improved reaction time in older persons and fluid intelligence in both age groups, while leading to a working memory decline in middle-aged users.

A concern when considering variable outcomes of studies of statin cognitive effects is weaknesses in study design, specifically a confounding effect through reverse causation and selection bias (extensively discussed in Power *et al*.^3^). Reverse causation could result from including subjects with already prevalent cognitive impairment or from drop-out of statin users that discontinued their medication. In both cases, the cognitive status of the individuals might have an influence on their statin use rather than their statin use having an influence on cognitive function. Also, in both cases, this could result in false positive results on statin beneficial effects or false negative results on statin detrimental effects. In order to avoid such bias, we only included subjects without disease background that could account for cognitive problems, and excluded statin discontinuers from the time of discontinuation. Although we cannot fully rule out an influential effect of statin discontinuation on our study outcomes, the low numbers of statin discontinuers (7.6% of the statin users followed up on at the second assessment and 5.9% of the statin users followed up on at the third assessment) as well as the observed diverse effects of statins on different cognitive functions in the two age groups make a fundamental bias rather unlikely. Selection bias arises in studies on statin effects through the markedly different health profiles of controls and statin users^3^. The common approach to overcome this issue is to include the possible confounders as covariates in the analysis. However, it should be clear that this approach has certain limitations, since it might not be comprehensive enough to assess the full health risk profile that impacts cognitive function. In this regard, we are currently working on a follow-up study, which specifically addresses the influence of common health risk factors of statin users on cognitive function. Still, our study has great clinical value as it demonstrates the differences in cognitive function of a common population of statin users compared to non-users, and the large amount of data available in the UK Biobank allowed us to include a large number of possible confounders in our model. By individually testing the influence of these parameters on the three cognitive functions of control subjects and statin users, we further ensured to only include important confounders and not decrease the power of our analysis by including unnecessary covariates.

We attempted to explore whether the differences in statin cognitive effects between the two age groups were related to treatment duration, as older statin users might have potentially used statins for a longer time than younger users. Our analysis did not reveal an effect of treatment duration on cognitive performance, however, this could be attributed to some limitations. First, the analysis had a very low resolution as the start of statin treatment was not known, and we could only stratify the subjects based on the information of whether or not they were taking statins at one of the three assessments. Due to this, we performed the analysis based on the third assessment, for which only a relatively small sample size was available. Thus, it is possible that our analysis of treatment duration was underpowered. Specifically designed randomized controlled trials, in which the cognitive performance of statin-naïve subjects is followed in close intervals from start of treatment, would give the possibility to draw further conclusions about possible temporal effects of statin treatment.

Very interestingly, in addition to the observed influential effect of age, our study suggests that statin cognitive effects vary with the specific cognitive function tested. If statins indeed differentially affect different cognitive functions, the use of different tests and test protocols in studies on statin cognitive effects might therefore add to the inconsistency of test results found in literature. Therefore, our results prompt further analysis of statin effects on distinct cognitive functions to validate our findings.

It might be worth noting that the vast majority (approximately 80%) of statin users included in our analysis were taking simvastatin. Thus, the effects we describe are to the most part attributable to simvastatin treatment. Although it is often assumed, and some indications exist, that lipophilic statins more easily cross over into the brain to affect neuronal function^12–14^, it is currently unclear if different types of statins affect cognitive functions differently, as study outcomes vary^15–17^. A general difficulty of assessing this issue is the limited number of hydrophilic statin users compared to lipophilic statin users, as it was the case in our study. In order to perform a conclusive, comparative study on the effects of lipophilic versus hydrophilic statins, it would be useful to have more detailed information on statin treatment duration than we had, as well as on the drug dosage and treatment compliance.

The exact mechanisms behind statin cognitive effects are at this time unresolved, but some general hypotheses exist that might explain beneficial as well as detrimental statin effects on cognitive function. Statins might achieve beneficial effects by ameliorating a series of deteriorative, age-related changes that increase the vulnerability and susceptibility to metabolic disorders^18^, leading to impaired brain energy metabolism^19^, accumulation of cholesterol^20^ and vascular damage^21^. These disturbances are highly linked to age-related cognitive decline as well as increase the risk for dementias and Alzheimer disease^22^. There are indications that the level of the Alzheimer disease-related β-amyloid is directly related to an increase in brain cholesterol levels^23^, and that β-amyloid accumulation accelerates the development of cognitive impairment^24–26^. Statins have been shown to improve metabolic function, reduce cholesterol levels and improve vascular health^27^, revealing possible mechanisms to explain beneficial effects on cognitive function during ageing. Detrimental effects of statins on cognitive function in younger persons might be the result of the same action, a reduction in brain cholesterol levels, but under these physiological conditions, disturbing cholesterol homeostasis and leading to a cholesterol deficit. In a related manner, statin-induced changes in brain cholesterol might affect myelination, and there are indications that statins improve white matter integrity in older people^28^, while it is conceivable that they might disrupt the myelination process in middle-aged individuals due to induction of a cholesterol deficit.

Finally, it is important to discuss the biological significance of the detected effects, as the study’s relatively large sample size provided strong statistical power. The effect of statin treatment on working memory and fluid intelligence might be considered rather small, as they appeared to have less impact on the cognitive performance compared to the effect of repeated testing and ageing. In contrast, the effect on reaction time among old patients was marked, and treated patients showed comparable performance to that of untreated patients which were up to 6 years younger.

In summary, our study indicates that user age might modify the effect of statins on different cognitive functions. Our results are intriguing, as they highlight the complex interactive nature of statin cognitive effects and give a possible explanation for the currently unresolved, discrepant study outcomes. Moreover, the findings warrant further analysis of the modulatory role of age on statin side effects, in order to improve current knowledge and shape guidelines for the use of statins in different age groups, as specifically statin users during midlife might need to be carefully assessed by clinicians.

## Methods

### Ethics

This study used the UK Biobank resource (http://www.ukbiobank.ac.uk/) to analyse data that has previously been collected. The UK Biobank study has been performed in accordance with the ethical standards laid down in the 1964 Declaration of Helsinki and its later amendments. All participants in the UK Biobank study have given informed consent for data collection, data storage and subsequent data analysis. Ethical permission for our study was given by the responsible local authorities, *Regionala Etikprövningsnämden* (now *Etikprövningsmyndigheten*, Swedish Ethical Review Authority, EPN), Uppsala, under registration number (*Diarienummer*, Dnr) 2017/198.

### Participants and data

The UK Biobank is a collection of longitudinal health-related data and biological samples from more than 500,000 voluntary participants in the UK that is available for research in the public interest. The initial sampling (*first assessment*) was conducted between 2006 and 2010. Around 20,000 participants were then followed up on during 2012 and 2013 (*second assessment*), and were further recalled for a third visit between 2015 and 2016 (*third assessment*).

We extracted data regarding the use of statins at all three assessments, plus information regarding sex, age, ethnic background, BMI, qualification, alcohol intake frequency, sleep duration, smoking status, physical activity, diagnosis of diabetes, high blood pressure, angina, heart attack, stroke, neurological and psychiatric diseases, medications for high blood pressure, cholesterol-lowering drugs, use of insulin and cognitive performance. The field IDs for all parameters used in this manuscript can be viewed in Supplementary Table S1.

Detailed information regarding the variables included in the study can be found on the UK Biobank website (http://www.ukbiobank.ac.uk/). The answers “I don’t know” and “Prefer not to answer” were recoded as missing. Education was recoded as binary, based on whether the participants did or did not hold a university/college degree. Ethnic background was assessed through an amalgam of sequential branching questions asked as part of the touchscreen questionnaire. Ethnic background was recoded as six top categories: white, black or black British, Asian or Asian British, mixed, Chinese, other. To assess smoking status, the participants were asked to indicate whether they were current smokers, previous smokers or had never smoked. To assess the frequency of alcohol intake, participants could choose one of six possible answers: never, on special occasions only, 1–3 times a month, 1–2 times a week, 3–4 times a week or almost daily/daily.

### Statins

We focussed our analysis on the use of lipophilic statins (i.e. atorvastatin, simvastatin and fluvastatin), as the number of persons using hydrophilic statins (i.e. rosuvastatin, pravastatin) was very small (7.0% hydrophilic statin users compared to 93.0% of lipophilic statin users at the first assessment). Of the lipophilic statin users, 79.0% used simvastatin, 20.7% used atorvastatin and only 0.3% used fluvastatin at the first assessment.

The UK Biobank did not include data on the duration of statin use for each participant at each assessment. However, data on the time of recruitment and follow-up as well as whether or not a person was using statins at these times was available, which enabled to code statin users into different treatment duration groups (see also description of *Modulatory influence of treatment duration on statin cognitive effects* further below).

For the main, longitudinal analysis (effect of statin use and age along the three assessments), individuals who were not on statins at the first assessment but started at a later assessment were included and moved from the control to the statin group from the time of treatment, while individuals who discontinued statin medication after the first assessment were excluded from the time of discontinuation. For the cross-sectional analysis (effect of treatment duration at the third assessment), controls contain only persons who never used statins throughout the entire assessment period, and individuals who were not on statins at the first assessment but started at a later assessment are included in the respective statin treatment duration group depending on the start of treatment.

### Cognitive readouts

We analysed the performance on a test of reaction time (card game “Snap”), working memory (“Pairs matching task”) and reasoning abilities (“Fluid intelligence test”), in order to assess the effect on different cognitive functions.

#### Reaction time

The participants’ reaction time was measured during 12 rounds of the card-game “Snap”. The participant was shown two cards at a time, and was instructed to press a button as quickly as possible if the two cards matched. The mean time (in milliseconds) to correctly identify matches is used as readout for reaction time in our study.

#### Working memory

In the “Pairs matching task”, the participants were presented with a set of cards and instructed to memorise the position of as many matching pairs of cards as possible. The cards were then turned face down on the screen, and the participant had to touch as many pairs as possible in the fewest tries. The first round used three pairs of cards, the second round used six pairs of cards. The number of incorrect matches in the second round was used as readout in this study. Individuals with a test duration equal to 0 deciseconds were excluded.

#### Fluid intelligence

The “Fluid intelligence test” measures the capacity to solve problems that require logic and reasoning ability independent of acquired knowledge. The participants had 2 minutes to answer as many questions as possible from a set of 13 questions. The readout used in our analysis was the number of correct answers*number of questions attempted/13) to receive a measure of success rate.

### Statistical analysis

The main statistical analyses were performed using the IBM Statistical Package for Social Science software (SPSS) version 24. Analyses of differences in health-related parameters between controls and statin users (based on UK Biobank output) as well as of training and age effects (based on adjusted means, standard errors of the means (SEMs) and sample sizes (Ns) from the main analysis) were performed with GraphPad Prism version 8.1.2. *P* values are reported down to *P* < 0.001. Graphs were prepared using GraphPad Prism version 8.1.2.

#### Inclusion/exclusion criteria

The UK Biobank baseline assessment concerned 502,543 individuals (Fig. 1). We included only people who reported a white ethnic background (n = 472,731), as it has been suggested that different ethnicities respond differently to statin medication^29,30^. We excluded people with a history of psychiatric (n = 5,776) and/or neurological (n = 27,982) disorders as diagnosed according to the ICD-10 criteria, to exclude prevalent cognitive impairment or dementia at baseline. Information regarding medication use was further available for 315,015 individuals at the first assessment. Based on this information, we included only lipophilic statin users in the statin group, as lipophilic and hydrophilic statins might act differently on cognition^15^. Due to a very small number of hydrophilic statin users, a separate analysis and comparison of lipophilic and hydrophilic statins was not possible in our study. At the individual assessments, we excluded people who reported to have been diagnosed with stroke (first assessment: n = 5,033, second assessment n = 87, third assessment: n = 38). We further excluded all individuals who had reported taking statins, but at the same time reported not being on cholesterol-lowering medications (first assessment: n = 2,377, second assessment: n = 56, third assessment: n = 54). On the other hand, controls who had reported being on cholesterol-lowering medication other than statins were included in the analyses, as the overall results were similar when excluding them. Finally, in the statin group, we excluded people who discontinued statin medication after the first assessment from the time of discontinuation (second assessment: n = 244, third assessment: n = 52), and, in the control group, excluded people who started statin treatment after the first assessment, from which time on they were included in the statin group (second assessment: n = 1,176, third assessment: n = 76). We did not specifically exclude outliers, but excluded all data sets containing missing values.

#### Differences in health-related parameters between controls and statin users

Differences between controls and statin users regarding population characteristics and physiological parameters, which might relate to statin treatment indication and cognitive function, were analysed using multiple t-tests, correcting for multiple comparisons within each parameter and age group using the Holm-Sidak method. The threshold for significance was further adjusted to account multiple testing: 0.05/4 (parameters) = 0.013. Differences regarding frequencies or disease incidences were analysed using the Fisher’s exact test. The threshold for significance was further adjusted to account for multiple testing: 0.05/10 (parameters) = 0.005. Differences in the alcohol intake score were analysed with Chi-square test. For this analysis, alcohol intake was ranked from 0–5 according to increasing intake frequency.

#### Covariate selection analysis

In order to reduce the number of covariates to be included in our final model, we tested the effect of each of the possible covariates on the cognitive scores of the three tests using generalised linear models (gamma model for reaction time, negative binomial with log link for working memory and linear model for fluid intelligence, robust estimation). This analysis was carried out separately for controls and statin users in order to detect possible interaction effects with statin treatment. We used data from the second assessment, and the statin users group only included people who were on statins on both the first and second assessment, to ensure a duration of treatment of at least one year. Only variables which were significantly (*P* < 0.05) associated with cognitive performance on either of the tests, and in either controls or statin users, were included in the final model.

#### Analysis of the general performance of controls and statin users in the three tests

The general performance of control subjects and statin users in terms of repeated assessment (training) effect and age effect (middle-aged versus old persons) was analysed using two-way ANOVAs and Sidak’s *post hoc* tests. A total of 15 (training effect) or 9 (age effect) individual ANOVAs were run, and the threshold for significance was adjusted accordingly to account for multiple testing in addition to the internal Sidak’s correction (training effect: 0.05/15 = 0.003; age effect: 0.05/9 = 0.006).

#### Longitudinal analysis of the effect of statin use and age on cognitive performance

In order to assess the general effect of statins on cognitive performance, we performed a longitudinal analysis over all three assessments. We used mixed-effect generalised linear models to assess the changes in cognitive performance over time in statin users versus controls and middle-aged (up to 65 years) and old (over 65 years) participants (gamma model for reaction time, negative binomial with log link for working memory and linear model for fluid intelligence, robust estimation). Time was set as random factor. All other factors were considered fixed factors. First, we tested whether an effect of statins existed on cognitive performance. Then, we checked for an interaction of statins and time as well as statins, time and age. The threshold for significance was set at P < 0.017 to correct for multiple testing (0.05/3 cognitive tests).

#### Cross-sectional analysis of the effect of treatment duration on cognitive performance

Second, we explored whether the duration of statin use had an impact on cognitive performance, by using the data from the third assessment. As previously mentioned, we had no information regarding the duration of use of statins, however we did have information regarding the time interval between the assessments for each individual. Therefore, the duration of statin use was estimated from the time interval between the individual assessments. Statin use was then coded in four categories: (1) individuals who did not report using statins at any of the three assessments (*controls*, no statin use, n = 1,105); (2) individuals reporting using statins only at the third assessment (*short-term users*, duration of statins use between 1 and 4 years; n = 76); (3) individuals reporting not using statins at the first assessment, but using statins afterwards (*medium-term users*, duration of statin use between 5 and 10 years, n = 152); (4) individuals who consistently reported being on statins at all assessments (*long-term users*, duration of statin use over 10 years, n = 262). To this aim, we used separate generalised linear models for each cognitive test (gamma model for reaction time, negative binomial with log link for working memory and linear model for fluid intelligence, robust estimation), corrected for the same covariates as in the longitudinal analysis. Additionally, we tested for the interaction between duration of statin use and age. The threshold for significance was set at *P* < 0.017 to correct for multiple testing.

## Supplementary information

Supplementary Table S1.

## References

[1] Yebyo HG, Aschmann HE, Kaufmann M, Puhan MA (2019). Comparative effectiveness and safety of statins as a class and of specific statins for primary prevention of cardiovascular disease: A systematic review, meta-analysis, and network meta-analysis of randomized trials with 94,283 participants. Am. Heart J..

[2] Wallach-Kildemoes H, Stovring H, Holme Hansen E, Howse K, Pétursson H (2016). Statin prescribing according to gender, age and indication: what about the benefit-risk balance?. J. Eval. Clin. Pract..

[3] Power MC, Weuve J, Sharrett AR, Blacker D, Gottesman RF (2015). Statins, cognition, and dementia—systematic review and methodological commentary. Nat. Rev. Neurol..

[4] Schultz BG, Patten DK, Berlau DJ (2018). The role of statins in both cognitive impairment and protection against dementia: a tale of two mechanisms. Transl. Neurodegener..

[5] Smeeth L, Douglas I, Hall AJ, Hubbard R, Evans S (2009). Effect of statins on a wide range of health outcomes: A cohort study validated by comparison with randomized trials. Br. J. Clin. Pharmacol..

[6] Arvanitakis Z (2008). Statins, incident Alzheimer disease, change in cognitive function, and neuropathology. Neurology.

[7] Szwast SJ (2007). Association of statin use with cognitive decline in elderly African Americans. Neurology.

[8] Beydoun MA (2011). Statins and serum cholesterol’s associations with incident dementia and mild cognitive impairment. J. Epidemiol. Community Health.

[9] Bernick C (2005). Statins and cognitive function in the elderly: The Cardiovascular Health Study. Neurology.

[10] Collins R, Armitage J, Parish S, Sleight P, Peto R (2002). MRC/BHF Heart Protection Study of cholesterol lowering with simvastatin in 20 536 high-risk individuals: A randomised placebo-controlled trial. Lancet.

[11] Anstey KJ, Lipnicki DM, Low L-F (2008). Cholesterol as a risk factor for dementia and cognitive decline: a systematic review of prospective studies with meta-analysis. Am. J. Geriatr. Psychiatry.

[12] Vuletic S (2006). Statins of Different Brain Penetrability Differentially Affect CSF PLTP Activity. Dement. Geriatr. Cogn. Disord..

[13] Botti RE, Triscari J, Pan HY, Zayat J (1991). Concentrations of pravastatin and lovastatin in cerebrospinal fluid in healthy subjects. Clin. Neuropharmacol..

[14] Saheki A, Terasaki T, Tamai I, Tsuji A (1994). *In vivo* and *in vitro* blood-brain barrier transport of 3-hydroxy-3-methylglutaryl coenzyme A (HMG-CoA) reductase inhibitors. Pharm. Res..

[15] Chu C-S (2018). Use of statins and the risk of dementia and mild cognitive impairment: A systematic review and meta-analysis. Sci. Rep..

[16] Sinyavskaya L (2018). Comparative effect of statins on the risk of incident Alzheimer disease. Neurology.

[17] Haag MDM, Hofman A, Koudstaal PJ, Stricker BHC, Breteler MMB (2009). Statins are associated with a reduced risk of Alzheimer disease regardless of lipophilicity. The Rotterdam Study. J. Neurol. Neurosurg. Psychiatry.

[18] Cutler RG (1991). Human longevity and aging: possible role of reactive oxygen species. Ann. N. Y. Acad. Sci..

[19] Yin F, Sancheti H, Patil I, Cadenas E (2016). Energy metabolism and inflammation in brain aging and Alzheimer’s disease. Free Radic. Biol. Med..

[20] Pallottini V (2007). Age-related HMG-CoA reductase deregulation depends on ROS-induced p38 activation. Mech. Ageing Dev..

[21] North BJ, Sinclair DA (2012). The Intersection Between Aging and Cardiovascular Disease. Circ. Res..

[22] Duron E, Hanon O (2008). Vascular risk factors, cognitive decline, and dementia. Vasc. Health Risk Manag..

[23] Wolozin B, Kellman W, Ruosseau P, Celesia GG, Siegel G (2000). Decreased prevalence of Alzheimer disease associated with 3-hydroxy-3-methyglutaryl coenzyme A reductase inhibitors. Arch. Neurol..

[24] Burns M, Duff K (2002). Cholesterol in Alzheimer’s disease and tauopathy. Ann. N. Y. Acad. Sci..

[25] Haley RW, Dietschy JM (2000). Is there a connection between the concentration of cholesterol circulating in plasma and the rate of neuritic plaque formation in Alzheimer disease?. Arch. Neurol..

[26] Frears ER, Stephens DJ, Walters CE, Davies H, Austen BM (1999). The role of cholesterol in the biosynthesis of beta-amyloid. Neuroreport.

[27] Liao JK, Laufs U (2005). Pleiotropic effects of statins. Annu. Rev. Pharmacol. Toxicol..

[28] Nadkarni NK (2015). Statins and brain integrity in older adults: secondary analysis of the Health ABC study. Alzheimers. Dement..

[29] Zissimopoulos JM, Barthold D, Brinton RD, Joyce G (2017). Sex and Race Differences in the Association Between Statin Use and the Incidence of Alzheimer Disease. JAMA Neurol..

[30] Mangravite LM, Thorn CF, Krauss RM (2006). Clinical implications of pharmacogenomics of statin treatment. Pharmacogenomics J..

