# Supplementary material for: The Cognitive Effects of Statins are Modified by Age
Source: Sci Rep. 2020 Apr 10;10:6187. doi: 10.1038/s41598-020-63035-2 (PMC7148321; doi:10.1038/s41598-020-63035-2)
Supplement: Supplementary file 1 — Supplementary Table S1. [file 41598_2020_63035_MOESM1_ESM.docx]

**THE COGNITIVE EFFECTS OF STATINS ARE MODIFIED BY AGE**

Ahmed M. Alsehli, Gaia Olivo, Laura E. Clemensson, Michael J. Williams, Helgi B. Schiöth

**Supplementary Table S1.** UK Biobank Field IDs

The field IDs for all parameters included in the manuscript are listed.

| Parameter | Field ID |
| --- | --- |
| Ethnic background | 21000 |
| Psychiatric or neurological disorders according to ICD-10 classification | 41202 |
| Medication use | 20003 |
| Age | 21003 |
| BMI | 21001 |
| Physical activity | 884 |
| Sleep duration | 1160 |
| Sex | 31 |
| Qualification Educations | 6138 |
| Diabetes | 2443 |
| Angina  Heart attack  Stroke  Hypertension | 6150 |
| Alcohol intake | 1558 |
| Smoking | 20116 |
| Reaction time | 20023 |
| Working memory | 399 |
| Fluid intelligence | 20016 |
